# Sleep matters: Neurodegeneration spectrum heterogeneity, combustion and friction ultrafine particles, industrial nanoparticle pollution, and sleep disorders—Denial is not an option

**DOI:** 10.3389/fneur.2023.1117695

**Published:** 2023-02-27

**Authors:** Lilian Calderón-Garcidueñas, Ricardo Torres-Jardón, Glen P. Greenough, Randy Kulesza, Angélica González-Maciel, Rafael Reynoso-Robles, Griselda García-Alonso, Diana A. Chávez-Franco, Edgar García-Rojas, Rafael Brito-Aguilar, Héctor G. Silva-Pereyra, Alberto Ayala, Elijah W. Stommel, Partha S. Mukherjee

**Affiliations:** ^1^College of Health, The University of Montana, Missoula, MT, United States; ^2^Universidad del Valle de México, Mexico City, Mexico; ^3^Instituto de Ciencias de la Atmósfera y Cambio Climático, Universidad Nacional Autónoma de México, Mexico City, Mexico; ^4^Department of Neurology, Geisel School of Medicine at Dartmouth, Hanover, NH, United States; ^5^Department of Anatomy, Lake Erie College of Osteopathic Medicine, Erie, PA, United States; ^6^Instituto Nacional de Pediatría, Mexico City, Mexico; ^7^Instituto Potosino de Investigación Científica y Tecnológica A.C., San Luis Potosi, Mexico; ^8^Sacramento Metropolitan Air Quality Management District, Sacramento, CA, United States; ^9^Department of Mechanical and Aerospace Engineering, West Virginia University, Morgantown, WV, United States; ^10^Interdisciplinary Statistical Research Unit, Indian Statistical Institute, Kolkata, India

**Keywords:** air pollution, Alzheimer's, nanoparticles, nanoneuropathology, PM2.5, sleep disorders RBD, OSA, depression

## Abstract

Sustained exposures to ubiquitous outdoor/indoor fine particulate matter (PM_2.5_), including combustion and friction ultrafine PM (UFPM) and industrial nanoparticles (NPs) starting *in utero*, are linked to early pediatric and young adulthood aberrant neural protein accumulation, including hyperphosphorylated tau (p-tau), beta-amyloid (Aβ_1 − 42_), α-synuclein (α syn) and TAR DNA-binding protein 43 (TDP-43), hallmarks of Alzheimer's (AD), Parkinson's disease (PD), frontotemporal lobar degeneration (FTLD), and amyotrophic lateral sclerosis (ALS). UFPM from anthropogenic and natural sources and NPs enter the brain through the nasal/olfactory pathway, lung, gastrointestinal (GI) tract, skin, and placental barriers. On a global scale, the most important sources of outdoor UFPM are motor traffic emissions. This study focuses on the neuropathology heterogeneity and overlap of AD, PD, FTLD, and ALS in older adults, their similarities with the neuropathology of young, highly exposed urbanites, and their strong link with sleep disorders. Critical information includes how this UFPM and NPs cross all biological barriers, interact with brain soluble proteins and key organelles, and result in the oxidative, endoplasmic reticulum, and mitochondrial stress, neuroinflammation, DNA damage, protein aggregation and misfolding, and faulty complex protein quality control. The brain toxicity of UFPM and NPs makes them powerful candidates for early development and progression of fatal common neurodegenerative diseases, all having sleep disturbances. A detailed residential history, proximity to high-traffic roads, occupational histories, exposures to high-emission sources (i.e., factories, burning pits, forest fires, and airports), indoor PM sources (tobacco, wood burning in winter, cooking fumes, and microplastics in house dust), and consumption of industrial NPs, along with neurocognitive and neuropsychiatric histories, are critical. Environmental pollution is a ubiquitous, early, and cumulative risk factor for neurodegeneration and sleep disorders. Prevention of deadly neurological diseases associated with air pollution should be a public health priority.

## 1. Introduction

Chronic exposures to outdoor concentrations of PM_2.5_ above WHO air quality guidelines (annual 5 μg/m^3^) caused 6.4 million premature deaths and 93 billion days lived with illness in residents worldwide in 2019 (1). Exposures to traffic-generated pollutants, residency close to high-traffic roads, incomplete combustion emissions, firepit emissions, and *in vitro* experimental PM exposures of neural tissues, among other sources, have all been associated with extensive neural damage and increases in neurodegenerative diseases, including AD, PD, and ALS for the last two decades (2–17). Millions of US residents are exposed to wild forest fires and live near high-volume traffic roads and traffic-related air pollution (TRAP) (18, 19). Disadvantage populations, including minorities and low-income individuals, are exposed to high TRAP pollution (19, 20).

This study focuses on how incomplete combustion species and friction-derived and industrial-sourced nanoparticles reach neural tissues and damage target organelles in the nervous system; how these UFPMs and NPs travel in the brain and affect brain hubs with extensive communications and key roles in the integration of critical information, including sleep (21–23). For this study, we would be using either UFPMs and/or NPs, since our focus is on particle size, i.e., ≤ 100 nm.

The identification of the initial neuropathological stages of Alzheimer's disease (hyperphosphorylated tau and amyloid beta) (24) in 202/203 Metropolitan Mexico City forensic autopsies, with an average age of 25.4 ± 9.2 years, including 44 children with an average age of 12.89 ± 4.9 years, and the progression of the disease by the second and third decades of life, along with the concomitant development of PD and TDP-43 pathology in young urbanites, are at the core of our research efforts and our deep interest in comparing sleep disorders in patients with AD, PD, FLTD, and ALS, the involvement of aberrant neural proteins, and the presence of UFPM and NPs in sleep hubs in young highly exposed to air pollution cohorts (9–11, 24–34).

Populations that are exposed chronically to high concentrations of outdoor and indoor PM_2.5_ are at higher risk of developing early diagnostic and neurodegenerative hallmarks, and the fact that they overlap from the earliest ages strongly suggests that there is a common denominator affecting the protein neural structures. UFPM and NPs could be the causative agents in association with genetic, epigenetic, and other environmental variables, and damaged sleep hubs, and resulting sleep disorders could be early findings (35–43).

Millions of people worldwide are exposed to outdoor and indoor environmental fine particulate matter (PM_2.5_) and nanosize PM ≤ 100 nm [ultrafine particulate matter (UFPM) and industrial nanoparticles (NPs)]. Metal combustion and friction-derived UFPM and NPs are identified in brain organelles starting *in utero* and are directly responsible for intense oxidative stress, protein misfolding, protein aggregation, and fibrillation. AD, PD, and ALS are associated with exposure to air pollutants. Sleep disorders are strong predictors of fatal neurodegenerative disorders.

## 2. Particulate matter pollution, what is it? How do we measure it? Why nanosize PM is key?

**Figure 1:**
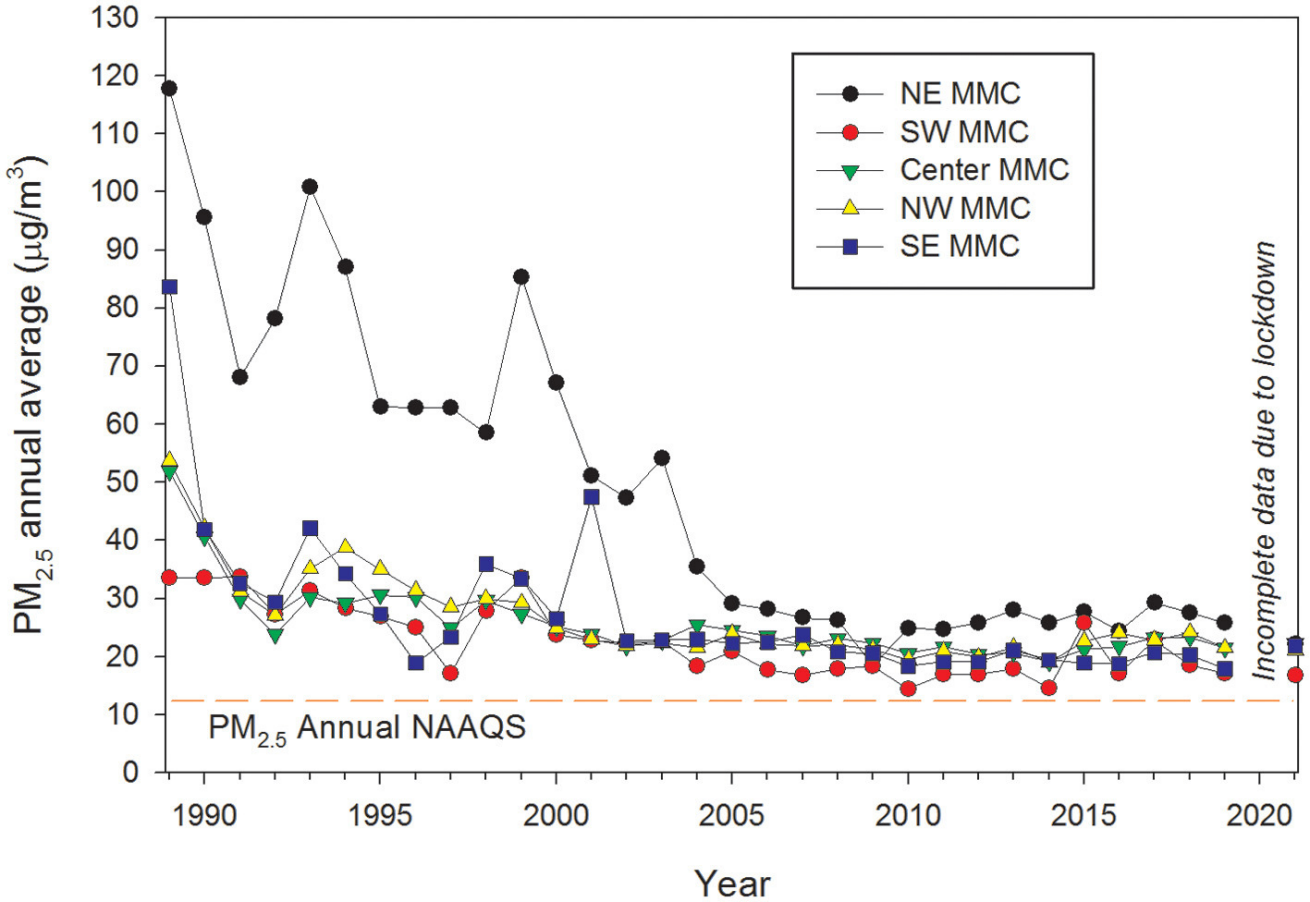
Time series trend of annual mean 24-h PM_2.5_ concentrations, averaged over 3 years, for five representative monitoring stations in MMC from 1990 to April 2020 and their comparison with the respective annual USEPA NAAQS. Data were processed and evaluated from measurements reported by the manual PM network of the Secretaría del Medio Ambiente del Gobierno de la Ciudad de México (SEDEMA) under a 6-day sampling schedule. Annual means from the years before 2004 were estimated from available information on PM_10_ since 1990 and the mean slope of the correlation PM_10_ vs. PM_2.5_ between 2004 and 2007. Source of data: http://www.aire.cdmx.gob.mx/default.php#.

Particulate matter (PM) consists of a mixture of microscopic solids and aerosols (liquid droplets) of different sizes and compositions found in the air. Different sizes of PM are based on their aerodynamic diameters: PM_10_ (mass of PM with an aerodynamic diameter <10 μm); fine or PM_2.5_ (particles <2.5 μm), and ultrafine particles (UFPM, with an aerodynamic diameter <0.1 μm). PM differs in chemical composition, size, shape, morphology, and air lifetime, depending mainly on their origin, which in turn can be primary or secondary. Particles emitted directly into the atmosphere are primary PM, while those formed within the atmosphere from a number of processes such as nucleation, condensation, and/or chemical reactions of gas-phase species are secondary PM, mainly gaseous air pollutants (44). PM_10_ and PM_2.5_ are our current indicators for PM pollution worldwide, particularly in highly polluted urban areas (i.e., Metropolitan Mexico City, Figure 1). Routine measurements of UFPM are neither common nor enforced, despite it being well-recognized that they can reach alveoli, circumvent primary airway defenses, and carry numerous toxic organic and inorganic compounds (44, 45).

Notably, while PM_10_ and PM_2.5_ ambient concentrations and their regulatory compliance with air quality standards are determined by mass-based methods, UFPMs have negligible mass, making them very difficult to measure. UFPMs are quantified by number concentration, which in many cases do not correlate with the mass concentrations reported as PM_10_ or PM_2.5_ (46–48). We currently do not have worldwide ultrafine particle matter regulations (21, 22). Although some countries have guidelines for UFPMs in terms of particulate number concentrations, their focus has been on short-term exposures in occupational environments and for specific materials, thus they do not apply to outdoor or indoor environments. Available measurement systems for UFPMs include condensation particle counters, electro-mobility spectrometers, diffusion battery counters, and photoelectric nucleus counters (44–49).

**Figure 2:**
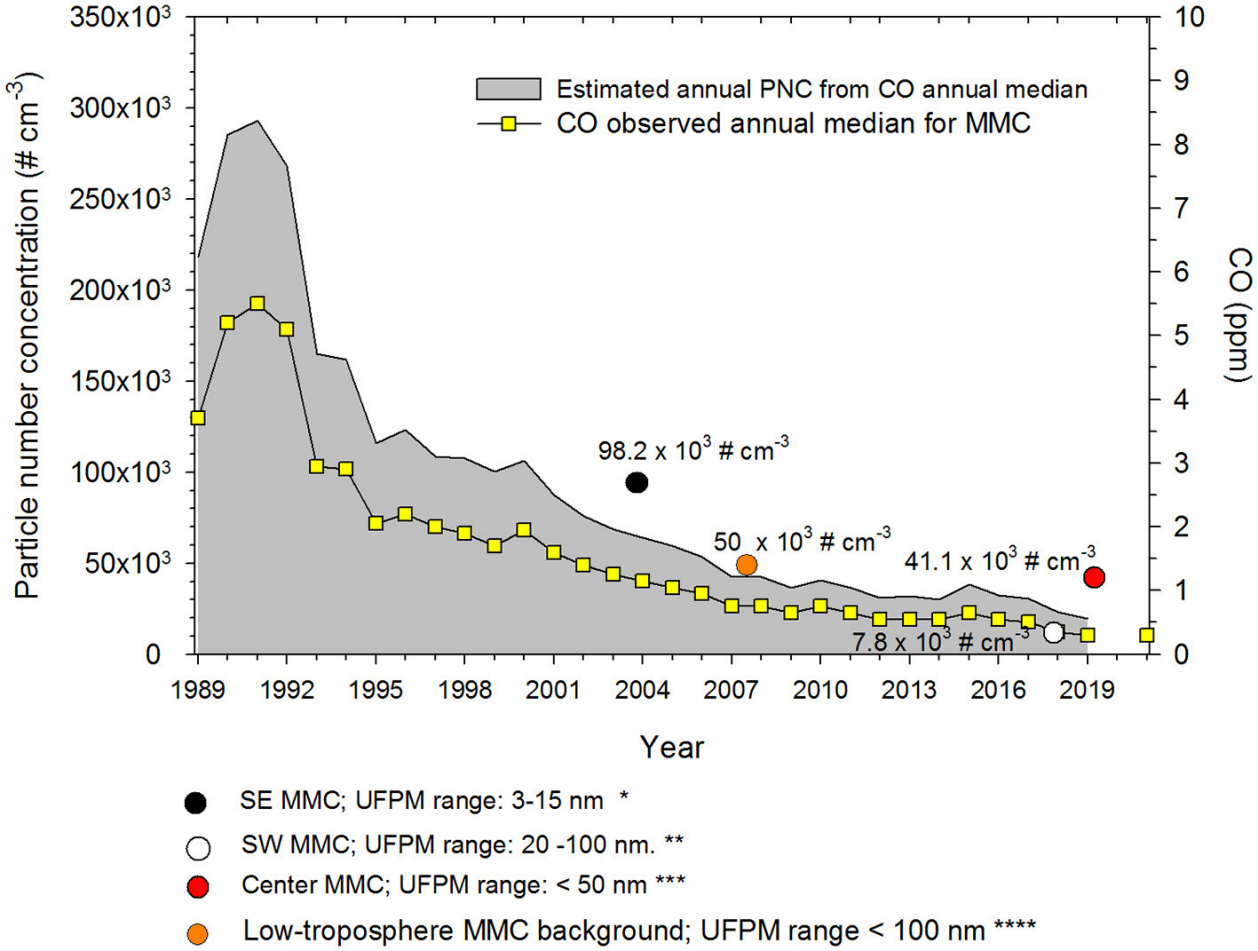
Trends of estimated PNCs and the associated annual medians of 1-h average CO for five representative monitoring stations of the MMC from 1989 to 2021. The colored circles in the figure correspond to the medians of PNCs measured by the authors referenced in (51*), (52**), (53***), and (54****). CO data source: http://www.aire.cdmx.gob.mx/default.php.

As road traffic and uncontrolled small combustion sources generate a significant number of nanoparticles, heavily polluted urban areas are suffering from strong UFPM problems (48). Metropolitan Mexico City (MMC) has experienced a dramatic increase in the number of vehicles in the last 20 years. Before 2000, CO and PM_2.5_ levels in MMC were among the highest levels registered in North America. However, due to actions to reduce traffic pollution, UFPM particle number concentrations (PNC) from the mid-2000s on, have been reduced to around 30,000 cm^−3^ (50). Using a non-linear correlation model between PNC, CO, and PM_2.5_ concentrations obtained from short-term monitoring studies, we have estimated that in the 1990s, PNC in MMC was around 300,000 cm^−3^ (50–54). Figure 2 shows the estimated annual average UFPMs number trend coupled with the CO annual median for MMC from 1989 to 2021 (50). We assumed that PM_2.5_ and CO could be reasonable proxies of vehicular emissions and incomplete combustion processes in the urban area. Typical particle number concentrations measured in 44 urban areas worldwide are in the order of ~5 × 10^3^ to ~8 × 10^4^ cm^−3^ with extremes above 1 × 10^5^ cm^−3^ in China and India (48).

## 3. Nanoparticles, metals, metalloids, and plastics. How harmful? How early? Where do they go in the brain? How relevant are systemic inflammation and neuroinflammation in neurodegeneration and their association with air pollution?

Nanoparticles, regardless of composition or shape, go everywhere in the body, cross all biological barriers, and go through paracellular pathways, including tight junctions, adherens junctions, and cytoskeletons (55–62). Their small size facilitates their absorption capabilities and their passage through membranes (5, 63–71); red blood cells (RBCs) and white blood cells (WBCs) are very efficient transporters of UFPM and NPs because they can reach any place, including the brain (12, 71). Their portals of entry (55) are key to understanding the importance of inhalation and ingestion of NPs and their direct brain entrance through the olfactory region and access to the trigeminal nerve. The inhalation entry starts in the nasal mucosa and continues to the alveolar space and the enormous lung capillary bed with the transport of UFPM and NPs through RBCs and WBCs and their free systemic circulation transportation. The massive amount of NPs we ingest every day have direct access to the intestinal epithelium and submucosa, causing significant damage to the paracellular structures and allowing direct entry of NPs to the enteric nervous system (ENS) (72).

The neurovascular unit (NVU) (73), defined as *a complex functional and anatomical structure integrated by endothelial cells, capillaries, arterioles, a basal lamina covered by pericytes, smooth muscle cells, and neural cells including neurons, interneurons, astrocytes, and an extracellular matrix*, is a direct UFPM/NP target, a critical observation explaining the extensive capillary and small arteriole vascular damage starting *in utero* and in childhood upon PM air pollution exposures (9, 10, 12, 55). As described by Schaeffer and Iadecola (73), NVU damage, regardless of the source, has serious effects on neurovascular regulation and coordination of vascular responses to central and peripheral signals, which are critical to maintaining brain homeostasis. NVU damage predicts neurodegeneration (21, 55, 72–74).

The detrimental impact of NPs on the brain includes high production of reactive oxygen species, neural inflammation, depletion of anti-oxidative enzymes, DNA damage, apoptosis, structural cell damage, including organelles, nuclei, tight junctions, adherens junctions, endothelial damage, and dysfunction (5, 55, 59, 62, 64, 70, 75–78).

Particularly relevant to this study is the fact that UFPM/NPs are very effective in their capacity to aggregate, conglomerate, and produce protein folding, destabilization, and fibrillation (5, 61, 63, 65, 67, 69, 70, 79–82). John et al. (81) referred to large nanostructures of ≥20 nm affecting the kinetic peptide aggregation, thus size and shape matter. They also discussed how NPs serve as a surface for the adsorption of peptide monomers and facilitate nucleation to oligomers and fibril formation (81). Mohammad-Beigi et al. (82) discussed how α-synuclein undergoes interactions with NPs and how these interactions can be prevented by the characteristics of the protein corona acquired during the exposure of NPs to serum proteins. When α-synuclein and polyethylenimine-coated carboxyl-modified polystyrene NPs (PsNPs-PEI) interact, the NP surface promotes the primary nucleation step of amyloid fibril formation, thus key to pathological fibrillation, serum proteins modulate the complex interplay between NPs and amyloid proteins (82).

NP/UFPM interactions with brain cells are complex, and variables such as the nature of the protein corona, bioavailability, biodistribution, size, shape, charge, composition, cell and organelle targets, and certainly portal of entry are all impacting the extent and type of brain damage.

**Figure 3:**
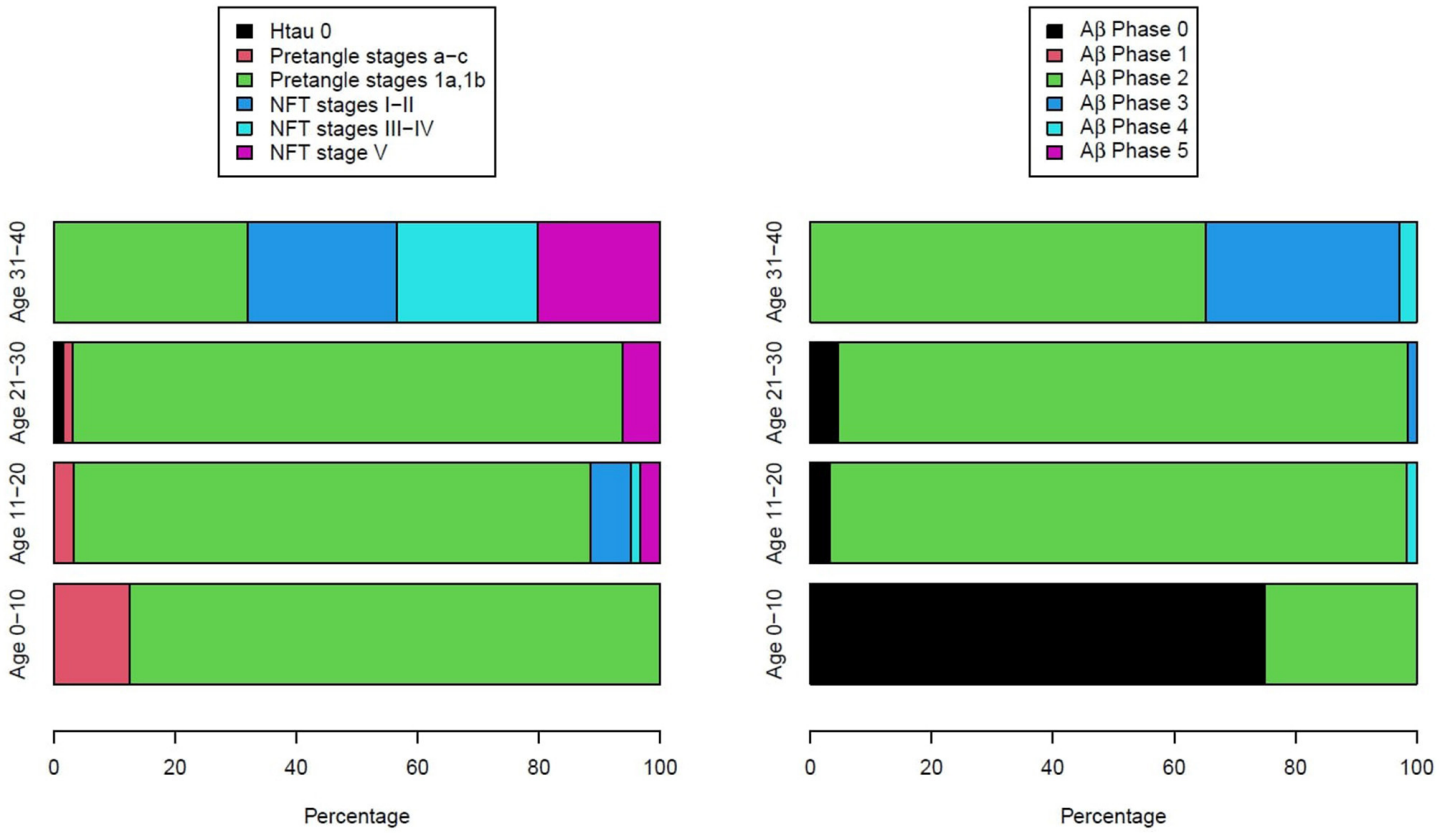
Two hundred and three forensic autopsies were staged for Alzheimer's disease using p-tau and Aβ_1 − 42_ (9). Subjects were 25.4 ± 9.2 years old and causes of death were related to car accidents, homicides, and suicides; 202/203 had AD pathology, including the youngest subject, an 11-month-old baby.

**Figure 4:**
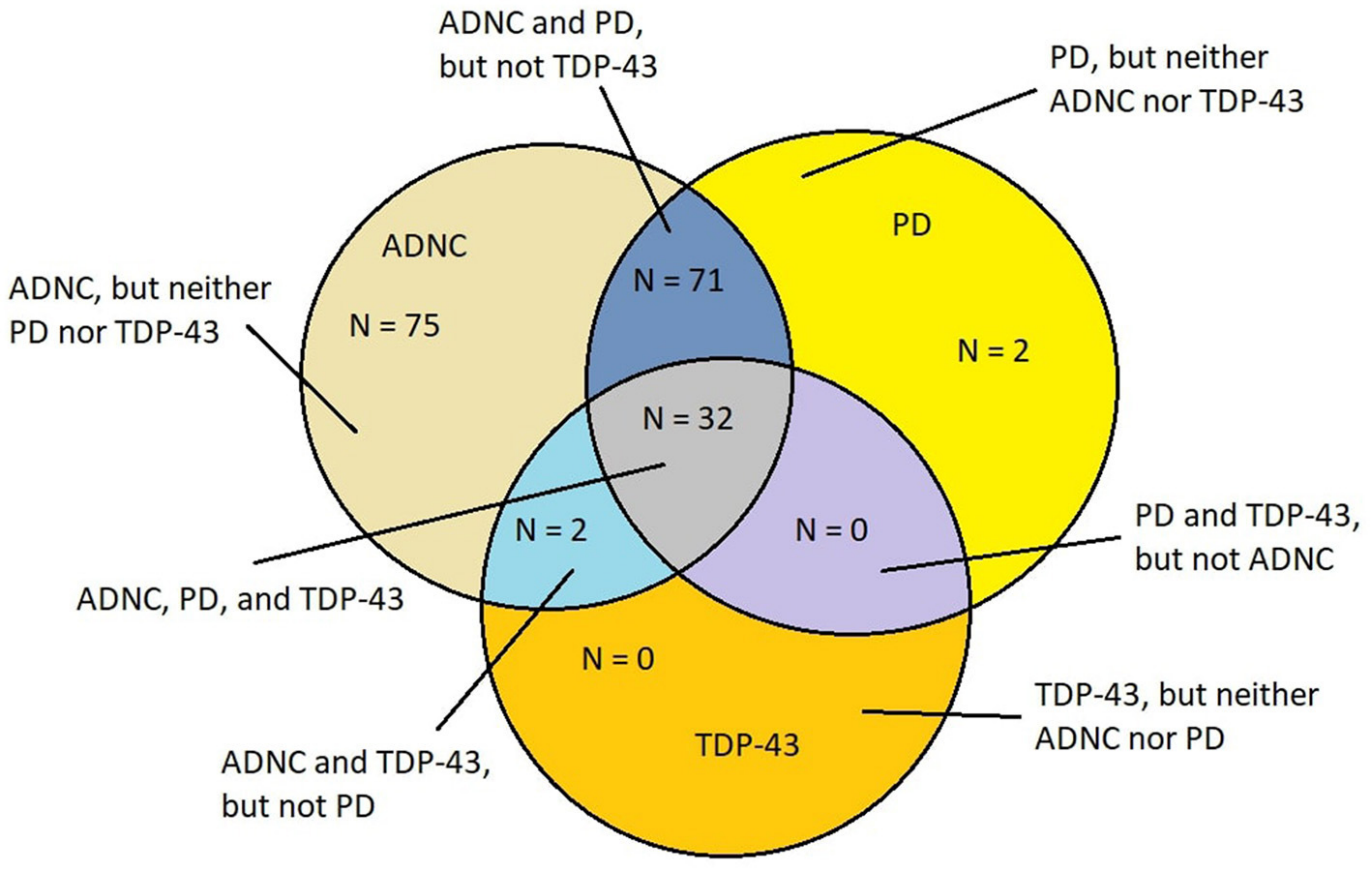
Venn diagram showing the overlap of neurodegenerative fatal diseases in a cohort of 186 MMC residents with an average age of 27.3 ± 11.8 years (10). Alzheimer's disease neuropathology changes (ADNC) (h-tau and Aβ) were present in each case.

An interesting and concerning factor in UFPM composed of iron (magnetite and maghemite) is precisely their magnetic properties (5, 83–85). In the study by Shu et al. (85), the superparamagnetic NPs could respond to an external magnetic field, and magnetic NPs could be seen setting down in the magnetic pole regions (see Figure 4d of that study). This magnetic cell settling does, in fact, occur in MMC residents, as we have documented the phenomenon in electron micrographs [(55), Figure 3B]. The issue is more than a sporadic finding; MMC brains contain significant concentrations of magnetic NPs measured as saturation remanent magnetization (SIRM), being highest in the cerebellum (10). The cerebellum in young MMC residents shows extensive vascular pathology and cerebellar endothelial erythrophagocytosis (50) and significant atrophy by volumetric brain MRI in young MMC residents (39).

Systemic inflammation and endothelial dysfunction are very relevant to air pollution exposures, as shown by our laboratory in Mexico City children (86), along with nasal inflammation, DNA nasal epithelial damage (87–90), and CSF inflammation (43). Systemic inflammation and endothelial dysfunction have been described in 295 pregnant women (91) with strong associations between increases in soluble vascular adhesion molecule-1 (sVCAM-1) levels for each 10 μg/m^3^ increase in PM_10_ concentration, strongly suggesting that inflammation and endothelial dysfunction have a key role in modulating the detrimental effects of air pollution exposure during pregnancy, as shown recently in our laboratory with the extensive presence of nanoparticles of industrial origin in placentas of all ages and brain fetal tissues (12).

Oxidative stress and inflammation are common denominators of particulate matter (PM) air pollution exposures (92), including PM containing polycyclic aromatic hydrocarbons (PAHs) at low exposure settings. Occupational exposures are equally important for both systemic and neural inflammation and neurodegeneration (93, 94). Orysiak et al. (93) described a significant increase in proinflammatory cytokines in firefighters, along with respiratory inflammation, a piece of information that is very significant given the massive exposure of the US population to forest fires (18) and traffic air pollution (19). Thus, the report of Huang et al. (94) on neuroinflammation in the 2001 World Trade Center (WTC) responders is not a surprise, nor is the increment in suicides among the same responder population (95–97). The expected responses of the highly PM-exposed WTC responders were precisely what researchers are publishing 22 years later and what we commented within hours of the tragic event: acceleration of neuroinflammation, neurodegeneration, and suicides, as we see in Mexico City residents, more pronounced in APOE4 carriers, and associated with dose and routes of exposure key for both WTC responders and MMC residents (9–12).

Monitoring systemic inflammation in children should be a health priority since ambient air pollution impacts inflammatory responses from childhood (86, 98). Certainly, UFPM/NPs play a key role in both systemic and neural inflammation (12, 14, 16, 17, 21, 45, 50, 55, 62, 64), and diesel and Fe-NPs cause significant damage to neural cells under experimental conditions (62, 64). The issue also applies to industrial NPs consumed worldwide in massive amounts, i.e., titanium oxide NPs (99). Rolo and coworkers (99) have an excellent review of the TiO_2_-NPs in foods causing *oxidative stress, cytotoxicity/apoptosis/cell death, inflammation, cellular and systemic uptake, genotoxicity, and carcinogenicity*, and although the authors made a plea to support limiting the use of TiO_2_-NPs in food, we are aware as toxicologists that the food industry will be reluctant to follow-up on the recommendations. Thus, although the literature supports the multiple pathways UFPM/NPs are capable of causing systemic and neural damage through oxidative stress, neuroinflammation, mitochondrial function, neurodegeneration, *via* excessive activation of cellular prion protein signaling, hippocampal-impaired neurogenesis and synaptic plasticity, abnormal peptidomic responses, apoptosis, and necrosis (100–105), we still do not have NPs and UFPM regulations in the United States, and we need to establish clear correlations between PM exposures, neurodegeneration, and inflammation (106–109).

## 4. Development of Alzheimer's and Parkinson's diseases and TDP-43 pathology in children and young adult MMC residents. The diagnostic neural abnormal proteins are present and overlap from childhood and are key for the diagnosis of early sleep disturbances

In 2002, we described the association between the neuropathological hallmarks of Alzheimer's disease and air pollution exposures in our laboratory, stating: *Neurodegenerative disorders such as Alzheimer's may begin early in life with air pollutants playing a crucial role* (37). *Two* decades later, we have robust evidence to support this statement in populations exposed to high levels of PM_2.5_ and UFPM/NPs. Our studies demonstrate the development of AD, PD, and TDP-43 pathology starting in childhood, and it is corroborated clinically with the progressive cognitive deterioration, abnormal gait and equilibrium, brainstem-evoked auditory potentials, olfactory deficits, sleep abnormalities, and brain MRI cortical, subcortical, and cerebellar atrophy in seemingly healthy individuals (36–42). Low CSF concentrations of amyloid β _1 − 42_ and BDNF differentiate children exposed to MMC air pollution from low pollution controls (43).

We have identified p-tau, the presence of Aβ and α-synuclein, and abnormal TDP-43 expression in 202 MMC forensic autopsies from residents who died in accidents, homicides, and suicides aged 25.3 ± 9.2 years (9, 10). Extensive, early, and progressive neurovascular unit damage and key organelle ultrastructural pathology were associated with metal- and metalloid-rich UFPM/NPs, making solid UFPM/NPs an agent for brain pathology in MMC subjects (9–12).

Figure 3 illustrates the two key AD neuropathology markers, namely, hyperphosphorylated tau and beta-amyloid, in MMC residents per decade, including 44 children (9). We thoroughly studied the extra neural tissues and confirmed there were no gross and/or light microscopy abnormalities.

As seen in Figure 3, every child had h-tau pre-tangle stages in the 1st decade of life, and by the 2nd decade, we documented neurofibrillary (NFT) tangles I–V (24). Subjects in the 4th decade were clearly in NFT I–V stages, and pre-tangle stages could no longer be identified. In contrast, Aβ progressed slowly and remained in the early phases. Interestingly, in our autopsy studies (9, 10), apolipoprotein E allele 4 (APOE4) carriers of the strongest Alzheimer's disease genetic risk factor (110–113) had higher AD Braak stages and the highest risk for suicide associated with lower cumulative exposures to PM_2.5_ vs. APOE3 carriers. A finding in keeping with the literature regarding the higher risk of carrying two copies of ε4 allele increasing the AD risk up to 15-fold versus an APOE3 carrier in European ancestry subjects (114). APOE is a key protein in the equation of AD risk, neuroinflammation, oxidative stress, and metals (35, 112–114). The study by Tcw et al. (115), relating local APOE haplotype and the ε4-specific amino acid changes to important deficits in lipid metabolism dysregulation, glial activation, and inflammation, is of considerable interest in the setting of air pollution (9).

The overlap of AD, PD, and TDP-43 pathology is remarkable in young MMC residents as it is extraordinarily similar to the mixed protein pathologies described in elderly demented patients diagnosed with AD, FTLD, LBD, PD, ALS, and cerebral amyloid angiopathy (CAA), white matter rarefaction (WMR) pathology, and in the younger than 60-year patients who are AD demented (25–34, 116–119). Metha and Schneider (118) illustrated the overlapping neuropathology in a Venn diagram, which was further discussed by Jellinger (119, 120). It is clear that in elderly populations, AD is a heterogeneous disease, and co-pathologies (119), including LBD and TDP-43 pathology, and cerebrovascular lesions, are critical for the clinical picture, imaging, and laboratory results (25, 119–128). Figure 4 illustrates the aberrant neural protein overlap in MMC young without extra neural pathology (10).

**Figure 5:**
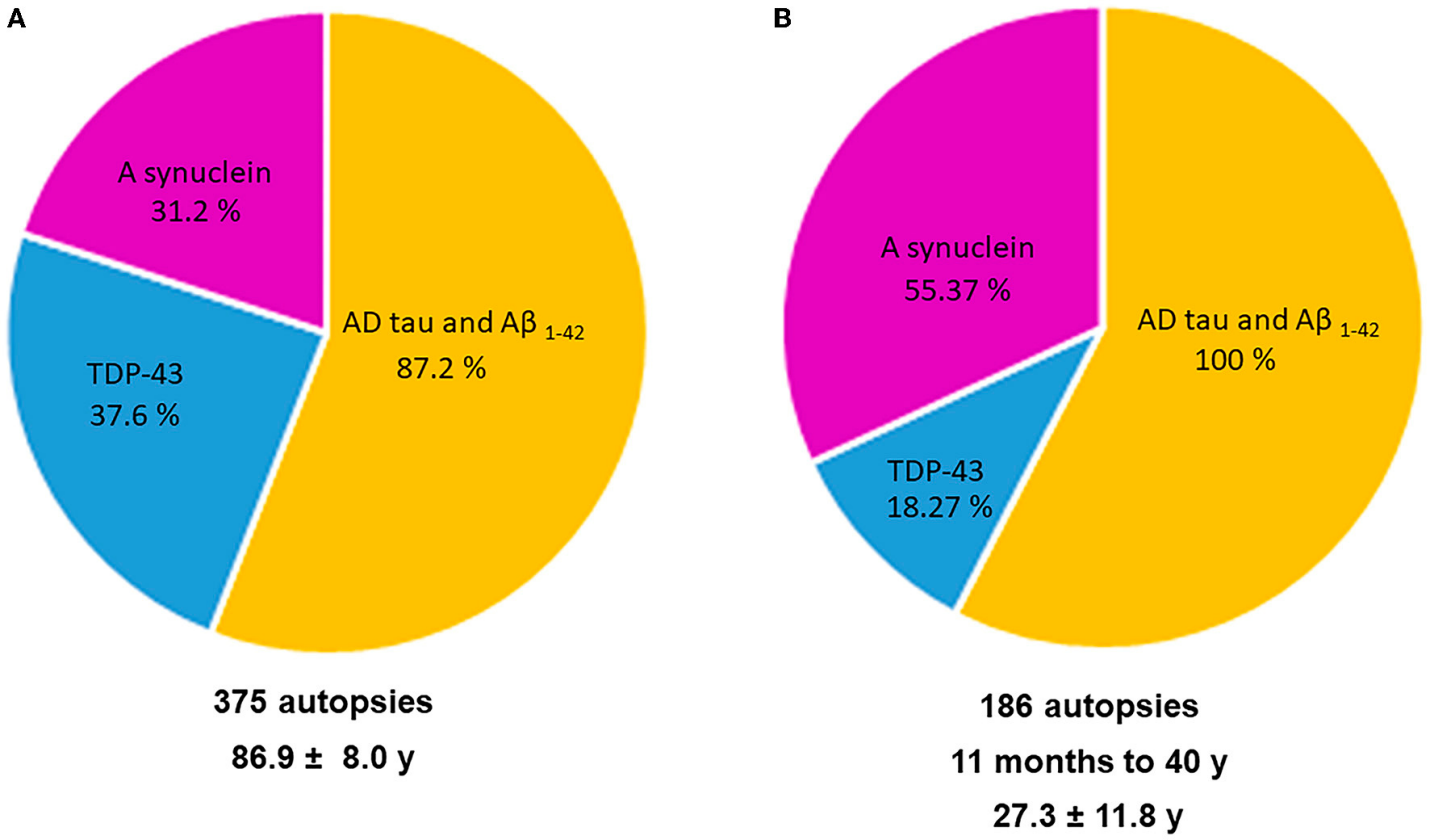
**(A, B)** A comparison in aberrant neural proteins between the young MMC 186 autopsy cohort (10) and Karanth et al. (25) 375 autopsies with an average age of 86.9 ± 8.04 years including subjects with normal cognition, mild cognitive impairment (MCI), impaired (but not MCI), and dementia.

All major neuropathological hallmarks of AD, PD, FTLD, and ALS are identified in young urbanites, from brainstem p-tau and diffuse amyloid plaques in an 11-month-old baby to extensive cortical p-tau in carriers of APOE4 alleles in 15-year olds. Common findings in MMC residents include p-tau in substantia nigrae and lack of nuclear TDP-43 in cortical motor neurons, lower motor neurons for cranial nerves III, V, and XII, and cervical motor neurons in teens and young adults (9–11). Hyperphosphorylated tau is definitely the major aberrant protein in highly exposed air pollution young urbanites (36). Figure 5 shows the overlap of aberrant neural protein in MMC young residents (10), compared to elderly subjects in the key work of Karanth et al. (25).

**Figure 6:**
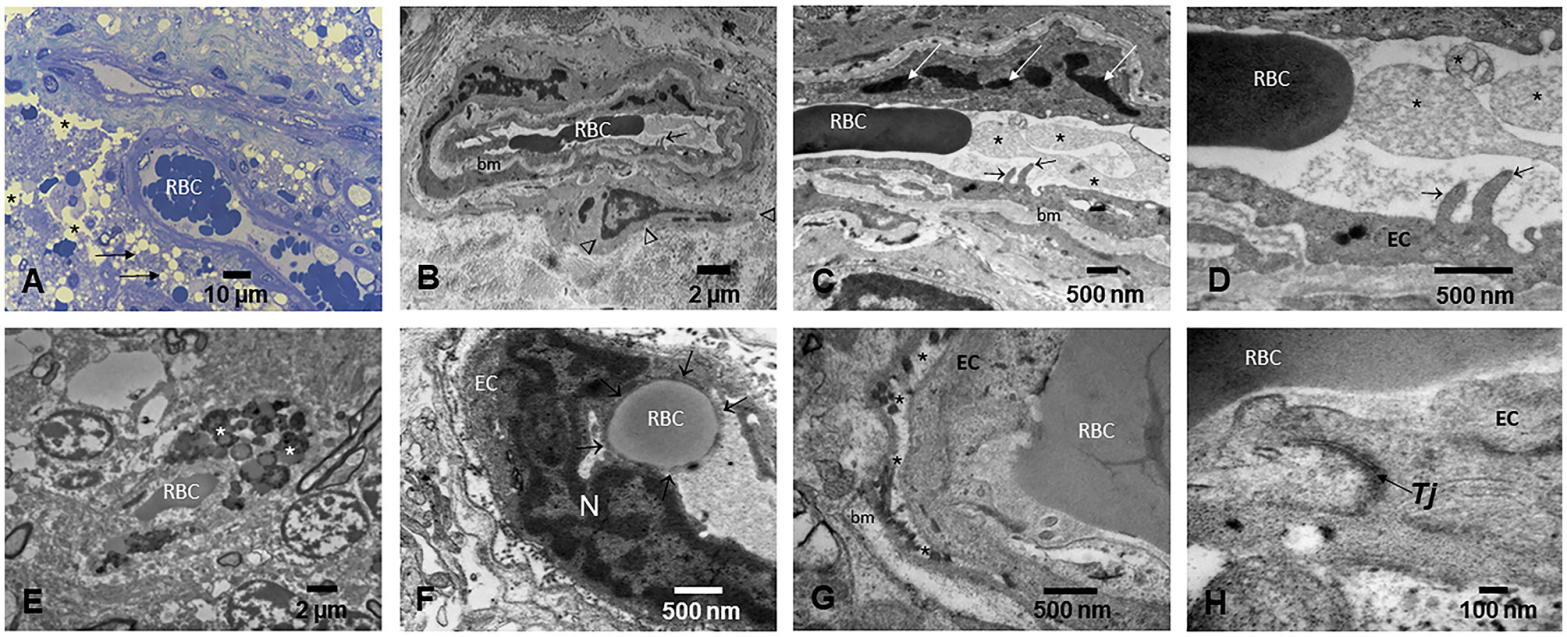
Light and electron microscopy of the brain in different anatomical locations in MMC young residents. **(A)** Thirteen-year-old girl's olfactory bulb showing small blood vessels with red blood cells (RBC) in the lumen and prominent endothelial cells. A significant number of perivascular vacuolated foamy cells (short arrows) and extensive areas of vacuolated neuropil ^(*^) are observed. **(B)** A frontal blood vessel showing two luminal RBCs and prominent endothelial extensions into the lumen (short arrow). The basement membrane (bm) is thickened, and a pericyte (arrow heads) is identified. **(C)** The activated endothelial cell sends filopodia into the lumen (short arrows), while the lumen is occupied by ghost cell fragments (*), seen in higher magnification in **(D)**. **(E)** Small frontal blood vessel containing one single luminal RBC and extravascular numerous lysosomal structures containing lipids and NPs (*). **(F)** Cerebellar blood vessel showing a typical RBC endothelial phagocytosis. The RBC is sequestered by the EC and surrounded by EC cytoplasm (short arrows). **(G)** Small blood cortical vessel with luminal RBCs closely in contact with the EC. The EC basement membrane is detached from the cell (*), and an accumulation of lysosome-like structures is seen. The basement membrane (bm) shows focal thickening. **(H)** A close-up of a tight junction Tj—a key structure in brain endothelial cells—showing poorly defined integrity (arrow).

Cerebrovascular pathology involving small and large cerebral vessels, with lesions ranging from gross and microscopic infarcts, atherosclerosis, and arteriolosclerosis, is commonly attributable to aging and independently associated with a higher risk of AD in elderly subjects (116, 118–120, 128). Strikingly, we have described extensive brain capillary and arteriole endothelial pathology and abnormal NVU in MMC dogs, children, and fetal brains in weeks 12–15 (12, 129). In dogs and children, capillaries displayed abnormal tight junctions—a critical component of the NVU (129), decorated with UFPM/NPs, and white matter extensive perivascular damage with leaking capillaries and arterioles displaying extravascular lipids and erythrocytes. The endothelial basement membranes are thickened and display beta-sheet structures, and the perivascular glial sheet is focally absent. NPs (10–48 nm) are localized in endothelial cells (EC), pericytes, and across the basement membranes. Endothelial damage associated with NPs is detected very early and worsens with age in children and teens with high PM exposures (Figure 6) (129).

We strongly suggest that neural abnormal protein overlap could be explained by the presence of UFPM/NPs in critical hubs with portals of entry, emission sources, cumulative exposures, size, shape, surface charge, chemical composition, biomolecular corona proteins, target organelles, cellular toxicity, axonal anterograde and retrograde transport, trans-synaptic movements, and a number of genetic (i.e., APOE4 carrier status) and environmental factors (*in utero* exposures), comorbidities, etc., accounting for the neural damage and the heterogeneity of neurodegenerative diseases (12–17, 25–34, 113, 116–129).

**Figure 7:**
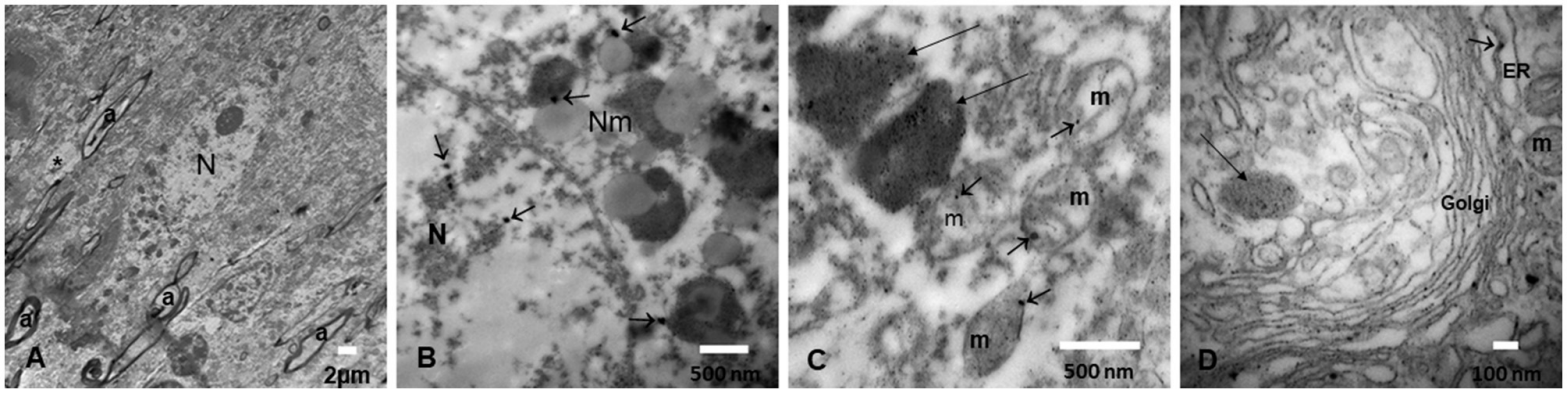
**(A)** Locus coeruleus (LC) neuron surrounded by loose neuropil (*) and myelinated axons (a). **(B)** An LC neuron with neuromelanin (Nm) structures containing nanoparticles (arrows) and similar NPs identified inside the nucleus (N). **(C)** Numerous NPs are identified both in large endolysosomal structures (long arrows) and in mitochondria (m) (short arrows). **(D)** The Golgi apparatus is a target of NPs, as well as the endoplasmic reticulum dilated structures (ER) (short arrows) and the lysosomal structures (long arrows).

The nanosize PM is composed of metals including Fe, Ti, Hg, Cu, Al, and Bi; post-transition metals, i.e., Al and Pb; alkaline earth metals, i.e., Ba; and non-metallic chemical elements such as Si are identified in every organelle in neurons, glial cells, microglia, and endothelial cells in Mexico City residents (10). Mitochondria and endoplasmic reticulum (ER), as well as the mitochondria-ER membrane contacts (MERC), are key NPs targets, and abnormal MERCs are common in highly exposed subjects (55, 56, 129). UFPM/NPs are also localized in the nuclear matrix—in close contact with heterochromatin—and nuclear pores. The outstanding accumulation of NPs in endolysosomes and specific structures like neuromelanin has great relevance in targeted neurodegenerative processes, including PD (Figure 7) (10, 55, 56, 130).

**Figure 8:**
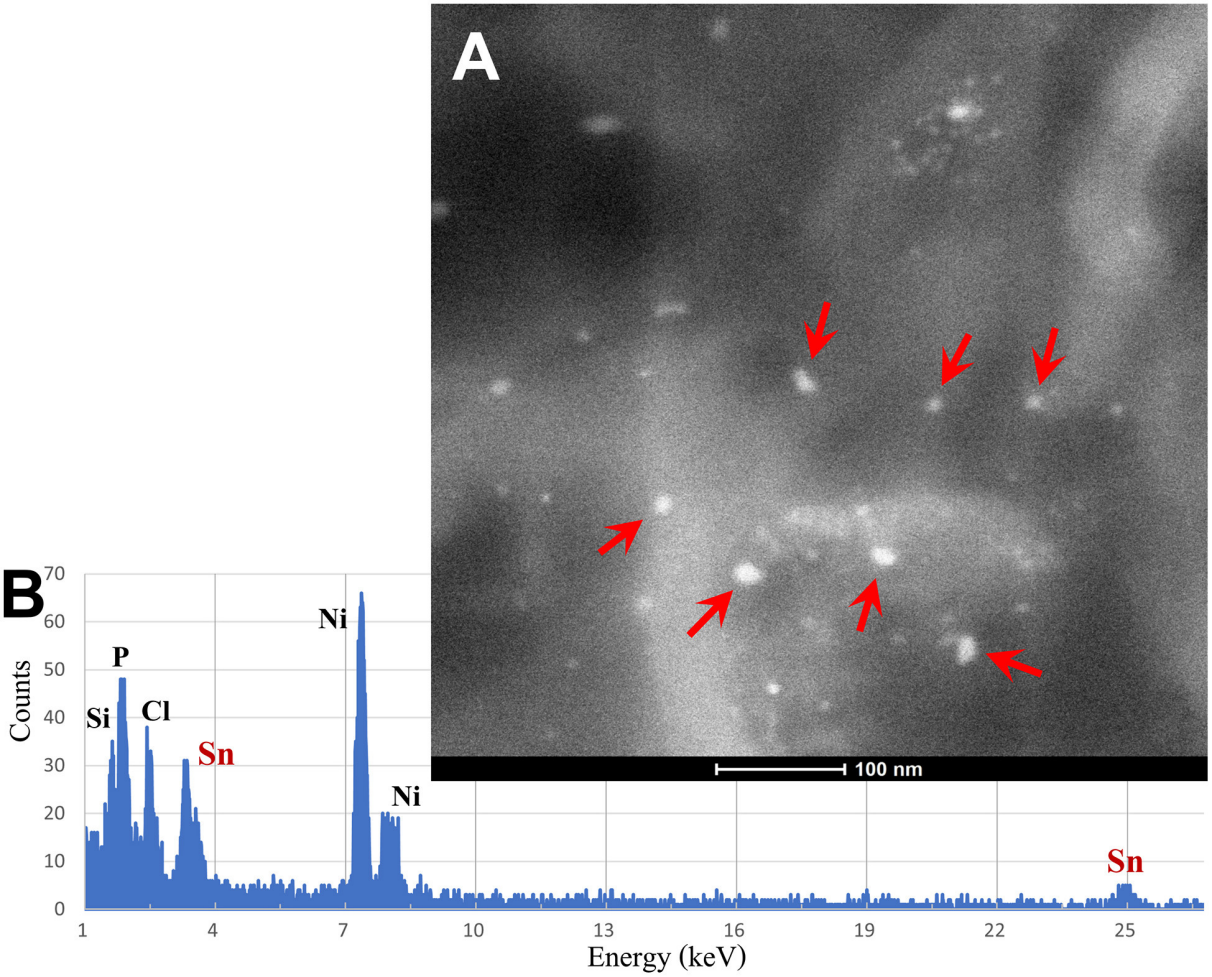
**(A)** Using the transmission electron microscopy (TEM) Z-contrast technique, metallic nanoparticles are documented in brain tissues. Only the nanoparticles marked with red arrows are Sn-NPs, while the rest are Fe-NPs. **(B)** The presence of Sn-NPs is verified through the acquired energy-dispersive X-ray spectrometry (EDX) that shows the tin metal (Sn) peak.

**Figure 9:**
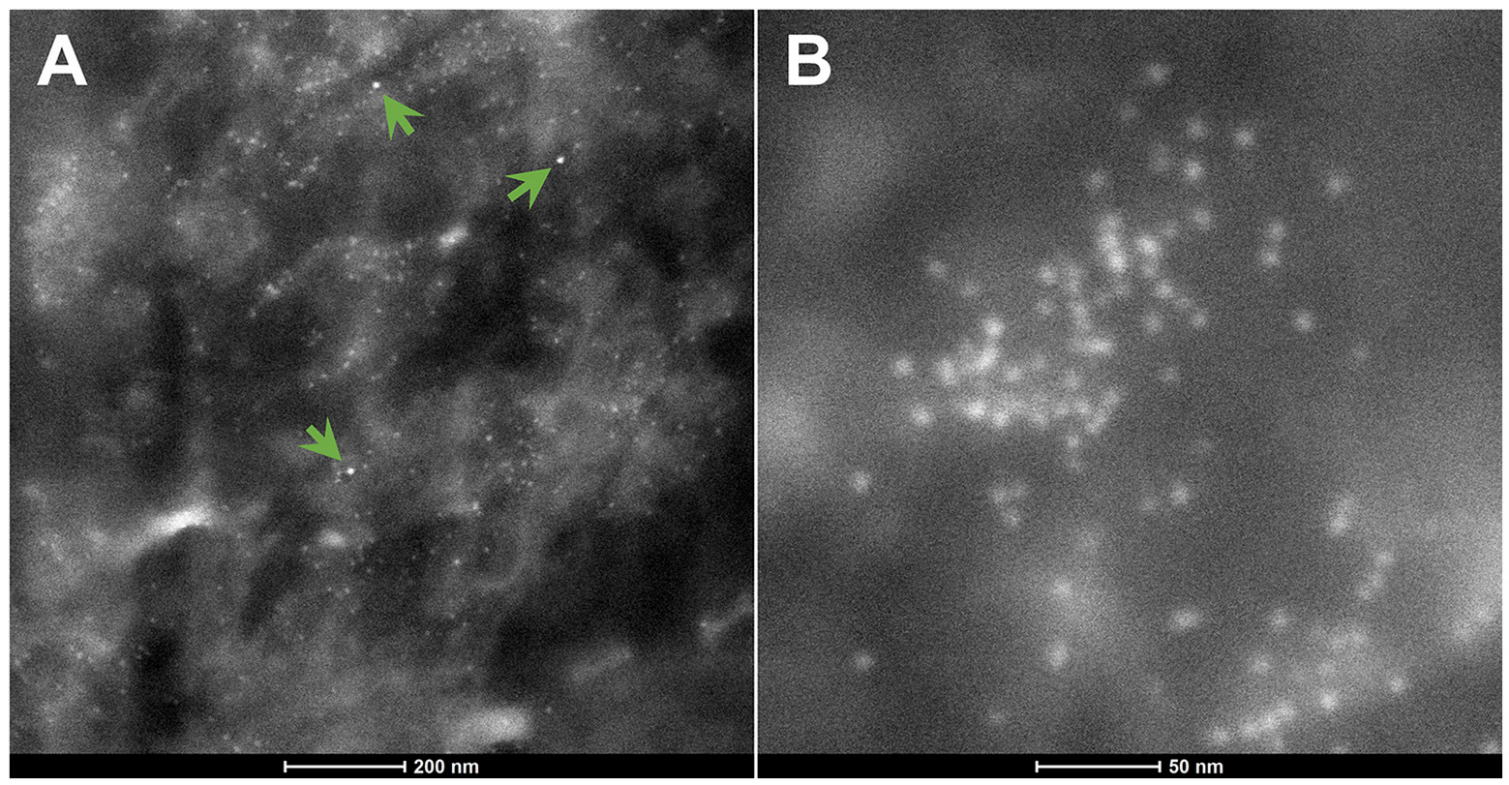
**(A)** The green arrows indicate three Hg-NPs that stand out from surrounding iron NPs. **(B)** An area of Fe-NPs. These NPs are magnetic.

The spectrum of metals and metalloids is critical for brain targets. We are identifying Fe-based, highly magnetic UFPM along with metals commonly associated with electronic waste, such as elongated TiO2 NPs (131). Shredding of e-waste is an extensive source of NPs in the United States (131), and very high concentrations of lead, for example, 2.9 μg-lead m^3^, are common 1.8 m away from the shredder operator, with extensive metal surface contamination reaching up to 250,000 particles cm^3^ with fine PM_2.5_ up to 171 μg m^3^, and both failing to return to background levels after 40 min of inactivity, as described by Ceballos et al. (131). As stated by Frazzoli et al. (132), *the aggressively extractive advanced technology industry thrives on the intensive use of non-renewable resources and hyper-consumeristic culture* and unfortunately, the health impact on the brain is detrimental. Figures 8, 9 show the metal and metalloid profiles in individual UFPM/NPs in neural and vascular cells analyzed by energy-dispersive X-ray spectrometry (EDX).

UFPM/NPs in targeted organelles with critical functions, including the assembly of proteins, lipid synthesis, regulation, transportation, clearing of damaged organelles *via* lysosomal degradation, inter-organellar communication, Ca^2+^ storage, transport and signaling, apoptosis, autophagy, stress responses, and formation and activation of inflammasomes, are at the core of the nanoneuropathology, as shown by the myriad of interesting studies focusing on alterations of mitochondria, MERCS, ERs, mitochondria-lysosome connections, neuromelanin, and nuclear pores (133–142).

Neurodegenerative diseases are heterogeneous, multisystem disorders with multiple abnormal proteins frequently associated with cognitive impairment and sleep disorders. The heterogeneity includes clinical-brain imagen variants that complicate diagnoses and putting forward we still have limitations for clinical and neuropathology diagnostic criteria (25–34, 116–129). AD, PD, and TDP-43 pathology start in childhood in populations with high exposures to PM_2.5_ (for this review, concentrations above the USEPA annual standards of 12 μg/m^3^) and UFPM and NPs.

## 5. Neurodegeneration spectrum heterogeneity, quadruple neural abnormal proteins, and sleep disorders

At the core of this study are the neuropathology spectrum heterogeneity and the overlap in neuropsychiatric outcomes, including sleep disorders (25–34, 117–128, 143–166). There is consensus that for specific sleep disorders, i.e., rapid eye movement sleep behavior disorder (RBD), the association with synucleinopathies, i.e., PD, LBD, or multiple system atrophy (MSA), is supported (143–146, 152–160, 162–166). RBD is regarded clinically as preceding the appearance of motor symptoms and cognitive decline by several decades and the overlap of neurodegenerative diseases is certainly present as magnifically shown by Boeve and collaborators (26). LBD, LBD and AD, MSA, AD, and progressive supranuclear palsy (PSP) were diagnosed at autopsy in patients with a clinical diagnosis of PD, cognitive impairment, and autonomic dysfunction (29). Further support for evidence of dopaminergic and cholinergic system alterations in neuroimaging is present in the literature (143, 144).

There is a complex etiopathogenesis involved in the association of sleep disorders and diseases such as PD. As discussed by Mizrahi-Kliger et al. (147), patients with PD exhibited at least four distinct pathways to explain their sleep problems: (i). a path directly associated with their PD synucleinopathy with regional involvement, (ii). medical therapy, (iii). degeneration of non-dopaminergic cells altering the circadian rhythm, and (iv). damage to brainstem dopaminergic neurons and its effect on the basal ganglia (147). Thus, common sleep complaints are linked to complex etiopathogenesis in the context of synucleinopathies, along with poor sleep quality associated with depression, PTSD, mood disorders, and excessive daytime sleepiness (148, 149, 156).

How much should we be concerned about sleep complaints and neurodegeneration? The answer is that we should be concerned depending on the patient, age, gender, clinical history, and how many risk factors, *including environmental factors*, are impacting neurodegenerative processes. The issue is relatively easy when we deal with a diagnosed elderly patient with AD or FTLD (151, 156, 157, 161), but not if we have a young adult resident in a polluted city or with occupational exposures (36).

It is in the younger adult population that learning about the etiopathogenesis of sleep disorders in AD, FTLD, PD, and movement disorders is helpful (24–34, 161, 167–169). Standlee and Malkani (169) underlined the mechanisms by which movement disorders are associated with sleep and circadian rhythm disruption, sleep fragmentation, insomnia, and excessive daytime sleepiness. It is worth emphasizing, as these authors did (169), the extensive involvement of brainstem nuclei regulating sleep and wakefulness in neurodegenerative processes.

At this time, it is unclear if sleep disturbances precede the common clinical neurodegenerative symptoms (i.e., cognition deficits) or if the sleep problems are some of the initial, early manifestations of neurodegenerative processes. Sleep complaints and neurodegeneration may be bidirectional. The sleep literature has addressed the abovementioned concerns in many different ways. For example, Zamore and Veasey (170) addressed chronic sleep disruption and neural damage, focusing on key variables, including duration and type of sleep disruption, age at which sleep loss occurs, neuronal populations responding to the injury, and the presence of genes involved in neurodegenerative processes. Sleep disruption impacts cognitive targets, such as episodic memory and sustained vigilance, pre- and post-synaptic impairment, the release of inflammatory cytokines and chemokines from microglia, and in transgenic 3×Tg-AD mice models, daily sleep-wake rhythm chronic fragmentation, increases in brain amyloid-beta (Aβ) levels, and neuroinflammation (170, 171). Grigg-Dambererger and collaborators (172) discussed acceleration of mild cognitive impairment (MCI) and dementia in patients with sleep-wake disorders and the removal of Aβ in non-rapid eye movement stage 3 sleep and fragmented or insufficient sleep leading to accumulation of abnormal neural proteins in preclinical stages. Burke et al. (173) explored the association between sleep disturbance and brain volumes in 1,533 subjects (cognitively normal/cognitively impaired or demented) using a single question from the Neuropsychiatric Inventory Questionnaire (NPI-Q): “Does the patient awaken you during the night, rise too early in the morning, or take excessive naps during the day?” The sleep disturbance was rated in a binary fashion (yes/no). Subjects with a yes answer to the NPI-Q question had a lower total brain, hippocampal volume and frontal and temporal lobe gray matter volume. The authors concluded as follows*: These findings suggest that disrupted sleep is associated with atrophy across multiple brain regions and ventricular hydrocephalus ex vacuo*. We will add that since the brain MRI findings take years to evolve, it is possible that the atrophic brain changes preceded the sleep disturbances. The direct relationship between subcortical wake-promoting damaged neurons and sleep phenotypes has been described by Oh et al. (174), in patients with AD and progressive supranuclear palsy (PSP). In fact, in 19 subjects, aged 70 ± 7.7 years at their demise, neuronal counts in three wake-promoting nuclei, namely, noradrenergic locus coeruleus [LC], orexinergic lateral hypothalamic area [LHA], and histaminergic tuberomammillary nucleus [TMN], were correlated with decreased homeostatic sleep drive. The authors suggested subcortical wake neurons correlate with sleep phenotypes in a number of neurodegenerative diseases, an observation that has practical and immediate applications (174).

On the other side of the relationship between sleep disorders and neurodegeneration, we find the literature on the brain impact of obstructive sleep apnea (OSA) and intermittent hypoxia as risk factors for preclinical AD and the incidence and progression of cognitive deficits (175–180). There is strong data on OSA producing intermittent hypoxia and sleep disruption and the observation that patients with OSA have higher serum levels of amyloid-beta and total tau and neuronal-derived Aβ and tau exosomes, going hand in hand with changes in sleep architecture (175, 176). There is strong evidence for the role of OSA as a risk factor for cognitive deficits (177) and the risk of developing or having OSA is significantly higher in patients with MCI or who are demented (178). It is also clear that several pathways are involved in neuropathological processes, including dysregulation of the orexinergic system and cerebral β-amyloid metabolism (179), major changes in CSF production, circulation, and glymphatic system abnormalities, which are crucial for the removal of metabolic waste (180). The American Thoracic Society workshop on the link between obstructive sleep apnea and neurocognitive impairment (181) concluded there is a strong biological plausibility but insufficient data to prove bidirectional causality of the associations between OSA and aging brain pathology. Thus, future research needs to address sleep disorders, oxidative stress, and accelerated brain aging (182, 183).

The WHO Mental Health Action Plan 2013–2030 emphasizes depression, affecting 4% of the population, ~280 million people, as an important cause of disability worldwide (184). Depression should be considered in the setting of sleep–wake disorders, anxiety, stress, burnout, and suicide (185, 186). Insomnia and mental health conditions coexist among US college students: depressed students (adjusted odds ratio, 9.54; 95% CI, 4.50–20.26) had significantly higher odds of insomnia, which were also significantly higher among employed students (odds ratio, 2.10; 95% CI, 1.05–4.18) (187). Sleep and mental disorders are related, and in the case of major depressive disorder (MDD), insomnia seems to be a comorbid disorder (188). The relationship between depression and sleep is highly concerning for sleep physicians given the association between depression and neuropathology (189). Villela Nunes and coworkers (189) examined the autopsies in 741 Brazilian non-demented individuals with an average age of 72.2 ± 11.7 years and major depressive disorder (MDD) (7.3%), late-life MDD (LLD) (10.8%), and depressive symptoms (DS) close to death (22.7%). Remarkably, all three correlated with small vessel disease: LLD and DS with brain infarcts and LBD, and DS with beta-amyloid plaques and amyloid angiopathy (189). Therefore, in fact, depression could be considered a premorbid neurodegeneration in elderly people (189), and it is associated with insomnia in young individuals (187).

The issues of sleep outcomes, sleep deprivation, sleep spindles, and neurodegeneration are critical to the bidirectionality of the relationship (190–194). The relationship between fast-frequency sleep spindles, aging, AD, and glial activation is very interesting and opens up the possibility of establishing an early marker associated with microglia dysfunction, synaptic loss, p-tau, and memory impairment. Sleep spindle deficits are a good example of the opportunity of using sleep variables as early AD biomarkers in aging and as trackers of AD progression (190–192). Furthermore, slow oscillations, sleep spindles, and their coupling during non-REM sleep are useful in experimental AD mouse models and could apply to patients with AD as key biomarkers and as guides to identify translationally relevant biomarkers and early intervention strategies to prevent or delay AD progression (193, 194).

Most of the associations between neurodegenerative processes have been done with particulate matter, especially UFPM and NPs, due to their capacity to travel to the brain and be localized in every organelle and cellular compartment (4, 5, 9–11, 16, 21, 22, 44, 137); however, the atmospheric chemistry is very complex and has to be seen as a continuum connecting emissions through chemistry and transport, as discussed by Finlayson-Pitts (195). Toxicologists and atmospheric chemistry researchers are working to understand sources, chemical characteristics, relationships between different pollutants, and transformations, as they are major challenges in air quality control and climate research (196, 197).

There has been a significant reduction in the solid fraction of PM in the United States and Europe; however, the generation of UFPM by nucleation of organic vapor during the dilution of the exhaust remains a serious issue (198), and the carbon UFPM from brakes, tires, and road wear will remain a problem even if we accomplish a fully electric vehicle fleet (198, 199). Furthermore, exposure to microplastics and nanoplastics is ubiquitous, and these nanoplastics can reach the brain and induce oxidative stress (57, 58).

Sleep is impacted in every neurodegenerative disease and has a robust link with depression. For a number of patients with sleep disorders, there is a close association between sleep complaints and the development and progression of well-characterized proteinopathies. It is important to determine if a sleep disorder is a consequence of the neurodegenerative process or if it plays a key role in the development of the neurodegenerative process itself. The bidirectionally/interplay between sleep and neurodegeneration makes sleep a critical physiological process subject to study in young populations with high risk for neurodegenerative pathologies.

## 6. Summary

1. Sleep disorders are common in neurodegenerative diseases, and the presence of targeted sleep problems associated with a high risk of development of common proteinopathies, along with significant associations between sleep deprivation, obstructive sleep apnea, intermittent hypoxia, cognitive deficits, pre-clinical AD, and other neurodegenerative pathologies, make sleep and neurodegeneration a focus for exploration in a number of patients sent to sleep laboratories (200).

2. Sustained and significant exposures to high concentrations of PM_2.5_ and UFPM/NPs are likely to play a significant role in the developing of neurodegenerative processes, dating back to *in utero* exposures. The presence of quadruple abnormal neural proteins starting in MMC infants and progressing as the subjects remain in the polluted environment should be of deep concern for health workers and has serious implications, including sleep disorders, for millions of people residing in such places.

3. We have shown the overlap of AD, PD, and TDP-43 pathology in highly exposed Mexico City children and young adults and the similarity of the overlap five decades later, when the patients are in terminal stages. We support that nanosized PM plays a key role in brain protein alterations and the complex subsequent cellular pathology.

4. Sleep disorders affect individuals of all ages with serious consequences across professions, including sleep deprivation in physicians (201). Our children are sleeping less, and there is a strong association between adverse childhood experiences and age-specific insufficient sleep duration in US youth, with serious repercussions in adulthood (202–208). There are also significant differences in sleep duration for US children depending on ethnicity and socioeconomic status (SES): among 9–13-year olds; black children sleep fewer hours compared to white, and poor children compared to higher-income children (204). Across the US, children sleep much less than what pediatricians recommend according to age, and minorities and disadvantaged children accumulate risk factors detrimental to their health (205). Moreover, lack of sleep increases the risk for addiction in adolescents based on chronic sleep loss and circadian misalignment (208). A potential association between inadequate sleep duration and changes in telomere length raises significant concerns related to cellular function (209).

5. The relationship between air pollution, sleep, neurodegeneration, depression, and suicide (210, 211) should encourage health workers to know about combustion and friction UFPM sources and engineered NPs (food products, cosmetics, toothpaste, sun protectors, surface disinfectants, paints, and e-waste). The presence of zinc, silver, copper, gold, selenium, and calcium NPs as potential food additives for animals (212), nanoplastics in drinking water (213), the massive presence of nanometric particle fraction of TiO_2_ in the food industry, and Fe_3_ O_4_ magnetic nanoparticles from food production, processing, storage, and detection, make constant exposures to NPs a serious health issue (76, 214).

6. The problem of human exposure to ultrafine particle pollution is solvable. We are knowledgeable of the cellular effects under experimental conditions and their intracellular and key organelle presence in the brains of urbanites (5, 10, 45, 48, 57–59, 62–71, 81, 82, 84). We also know the main emission sources and the technological options to control them (27, 215–217). The cost-benefit ratio is in favor of raising awareness (the role of our study) and taking action. We need a broader concern and awareness and the will to protect public health from deadly UFPM and industrial nanoparticles. We are also facing a lack of support for research from sleep medical societies. Denial is not an option.

## Author contributions

LC-G, RT-J, and PM: conception and design of the study. LC-G, RT-J, PM, GG, RK, AG-M, RR-R, GG-A, DC-F, EG-R, RB-A, and HS-P: acquisition and analysis of data. LC-G, AA, RK, PM, ES, and GG: drafting of the text, writing, critical analysis, and preparing the figures. PM: statistical analysis. RT-J: air pollutant data. All authors contributed to the article and approved the submitted version.
